# Electrocardiographic and Hemodynamic Changes During Tilt-Table Testing in Patients With Syncope

**DOI:** 10.7759/cureus.108212

**Published:** 2026-05-04

**Authors:** Ugne Stirkaite, Diana Rinkuniene

**Affiliations:** 1 Medical Academy, Lithuanian University of Health Sciences, Kaunas, LTU; 2 Cardiology, Lithuanian University of Health Sciences, Kaunas, LTU

**Keywords:** autonomic nervous system, electrocardiography, hemodynamics, orthostatic hypotension, syncope, tilt-table testing, vasovagal syncope

## Abstract

Background

Syncope is a common clinical condition with diverse underlying mechanisms, making its evaluation challenging. Tilt-table testing is widely used to assess autonomic and cardiovascular responses; however, the role of electrocardiographic (ECG) parameters in differentiating syncope types remains unclear.

Methodology

This retrospective study analyzed patients who underwent tilt-table testing for syncope evaluation in 2023-2024. A total of 59 patients were included and divided into three groups: orthostatic hypotension (n = 20), vasovagal syncope (n = 19), and negative test results (n = 20). ECG parameters (P-wave duration, PR interval, QRS duration, QTc interval, and T-wave duration) and hemodynamic variables (systolic and diastolic blood pressure and heart rate) were assessed at five time points. Linear mixed models were used to evaluate the effects of group, time, and their interaction.

Results

Most ECG parameters showed significant changes over time; however, no consistent group-time interactions were observed, indicating similar temporal patterns across groups. P-wave duration and PR interval differed between groups. In contrast, both systolic and diastolic blood pressure demonstrated significant group-time interactions (p < 0.001), reflecting distinct hemodynamic response patterns between syncope types. Heart rate increased significantly during orthostatic testing but did not differ between groups.

Conclusions

ECG parameters reflect general autonomic adaptation but lack specificity for differentiating syncope etiology. In contrast, hemodynamic responses, particularly blood pressure changes, provide greater diagnostic value and should be the primary focus during orthostatic testing.

## Introduction

Syncope is a common clinical problem characterized by a transient loss of consciousness due to global cerebral hypoperfusion [1]. It represents a significant diagnostic challenge, as its underlying causes range from benign reflex-mediated mechanisms to potentially life-threatening cardiovascular conditions. Accurate identification of the etiology is essential for appropriate management and risk stratification [2].

Tilt-table testing is commonly used in clinical practice for the evaluation of patients with unexplained syncope. It allows assessment of autonomic and cardiovascular responses to orthostatic stress and plays a key role in distinguishing between different syncope mechanisms, such as orthostatic hypotension (OH) and vasovagal syncope (VVS) [3-5]. Traditionally, the interpretation of this test relies primarily on hemodynamic parameters, including blood pressure and heart rate changes [2,5]. In addition to hemodynamic responses, electrocardiographic (ECG) parameters may provide further insight into cardiac electrophysiological adaptations during orthostatic stress. Previous studies have suggested that ECG intervals, reflecting atrial conduction, atrioventricular conduction, and ventricular repolarization, may change during tilt-table testing [6-9]. However, their clinical utility in differentiating syncope types remains unclear.

Given the complex interaction between autonomic regulation, hemodynamic responses, and cardiac electrophysiology, a comprehensive evaluation of both ECG and hemodynamic parameters during orthostatic testing may improve the understanding of syncope mechanisms. Previous studies have evaluated combined ECG and hemodynamic responses during tilt-table testing; however, these investigations have primarily focused on heart rate or selected hemodynamic parameters rather than detailed ECG interval analysis. Moreover, simultaneous longitudinal comparison of electrocardiographic and hemodynamic parameters remains limited. Therefore, this study aimed to evaluate ECG and hemodynamic changes during passive orthostatic testing, with a primary focus on characterizing physiological trends, and to explore whether these parameters may help differentiate the underlying causes of syncope. We hypothesized that hemodynamic parameters would demonstrate greater discriminatory value compared to ECG parameters.

## Materials and methods

A retrospective, single-center study was conducted at the Hospital of Lithuanian University of Health Sciences Kaunas Clinics (Kaunas, Lithuania) to analyze the results of patients who underwent tilt-table testing for syncope evaluation between 2023 and 2024. Approval to conduct the study was obtained from the Bioethics Committee (approval number: BE-2-69).

Patients were selected based on the availability of high-quality ECG recordings suitable for analysis, representing a convenience sample. Patients with incomplete or poor-quality data were excluded, and no imputation for missing data was performed. Additional inclusion criteria included age ≥18 years, sinus rhythm at rest, and no evidence of arrhythmia or conduction abnormalities based on clinical records and baseline electrocardiograms. Patients not meeting these criteria were excluded. A total of 59 patients were included in the study. Based on the results of the tilt-table test, patients were divided into the following three groups: OH group (n = 20), VVS group (n = 19), and negative test result group (n = 20) [10].

Demographic data, including age, sex, and body mass index (BMI, kg/m²), were collected. The tilt-table test was performed for all patients according to a standard protocol. ECG parameters were assessed in standard lead II at the following five predefined time points: after five minutes in the supine position (baseline), after 10 minutes in the supine position (pre-tilt), three minutes after tilting (early tilt), 20 minutes after tilting or at the time of syncope (peak orthostatic response), and after returning to the supine position (recovery). At each time point, the following ECG parameters were evaluated: P-wave duration, PR interval, QRS duration, QT interval, and T-wave duration. ECG recordings were obtained at a paper speed of 25 mm/s, and intervals were measured manually using a Medtronic (Minneapolis, MN, USA) ruler. All measurements were performed by a single investigator. Interobserver and intraobserver variability were not assessed. In addition, systolic blood pressure, diastolic blood pressure, and heart rate were recorded at each time point. The corrected QT interval (QTc) was calculated using the Bazett formula.

Statistical analysis was performed using SPSS Statistics software version 31 (IBM Corp., Armonk, NY, USA). Descriptive statistics for demographic variables were presented as median and interquartile range (IQR). The chi-square test was used to assess associations between categorical variables. For comparisons of continuous variables between two independent groups, the Mann-Whitney U test was applied, while comparisons among more than two groups were performed using the Kruskal-Wallis test. When statistically significant differences were identified, Dunn-Bonferroni post hoc analysis was conducted. ECG parameters were analyzed using linear mixed models, including group, time, and their interaction as fixed effects, and subject as a random effect. Formal assessment of model assumptions was not performed. Age was not included as a covariate in the primary analysis. The level of statistical significance was set at an α of 0.05, and results were considered statistically significant when p-values <0.05.

## Results

Evaluation of demographic characteristics and baseline hemodynamic parameters revealed a significant difference in age between the study groups (p < 0.001). Post hoc analysis with Bonferroni correction showed that patients in the VVS group were significantly younger than those in both the OH group (p < 0.001) and the negative test group (p = 0.038), whereas no significant difference was observed between the OH and negative test groups (p = 0.088). No statistically significant differences were identified for the remaining variables (Table 1).

**Table 1:** Demographic characteristics and baseline hemodynamic parameters of the study population. Total sample size: n = 59. Categorical variables were compared using the chi-square test. Continuous variables were compared using the Kruskal–Wallis test and are presented as median (IQR). OH = orthostatic hypotension; VVS = vasovagal syncope; BMI = body mass index; SBP = systolic blood pressure; DBP = diastolic blood pressure; HR = heart rate; IQR = interquartile range

Characteristics	OH group (n = 20)	VVS group (n = 19)	Negative test group (n = 20)	Test statistic	P-value
Gender				χ² = 4.82	0.090
Women, n (%)	4 (20%)	10 (52.6%)	9 (45%)		
Men, n (%)	16 (80%)	9 (47.4%)	11 (55%)		
Age, years, median (IQR)	65 (52-73)	36 (28-41)	48 (42-63)	H = 21.54	<0.001
BMI, kg/m², median (IQR)	25.5 (24.3-29.5)	24.8 (22.6-25.9)	24.7 (21.4-26.9)	H = 3.07	0.215
Baseline SBP, mmHg, median (IQR)	134 (123-153)	121 (114-138)	130 (116-140)	H = 3.14	0.208
Baseline DBP, mmHg, median (IQR)	79 (70-88)	74 (61-85)	79 (73-85)	H = 2.06	0.358
Baseline HR, beats/minute, median IQR)	67 (63-76)	66 (62-77)	70 (64-78)	H = 1.24	0.537

**Figure 1:**
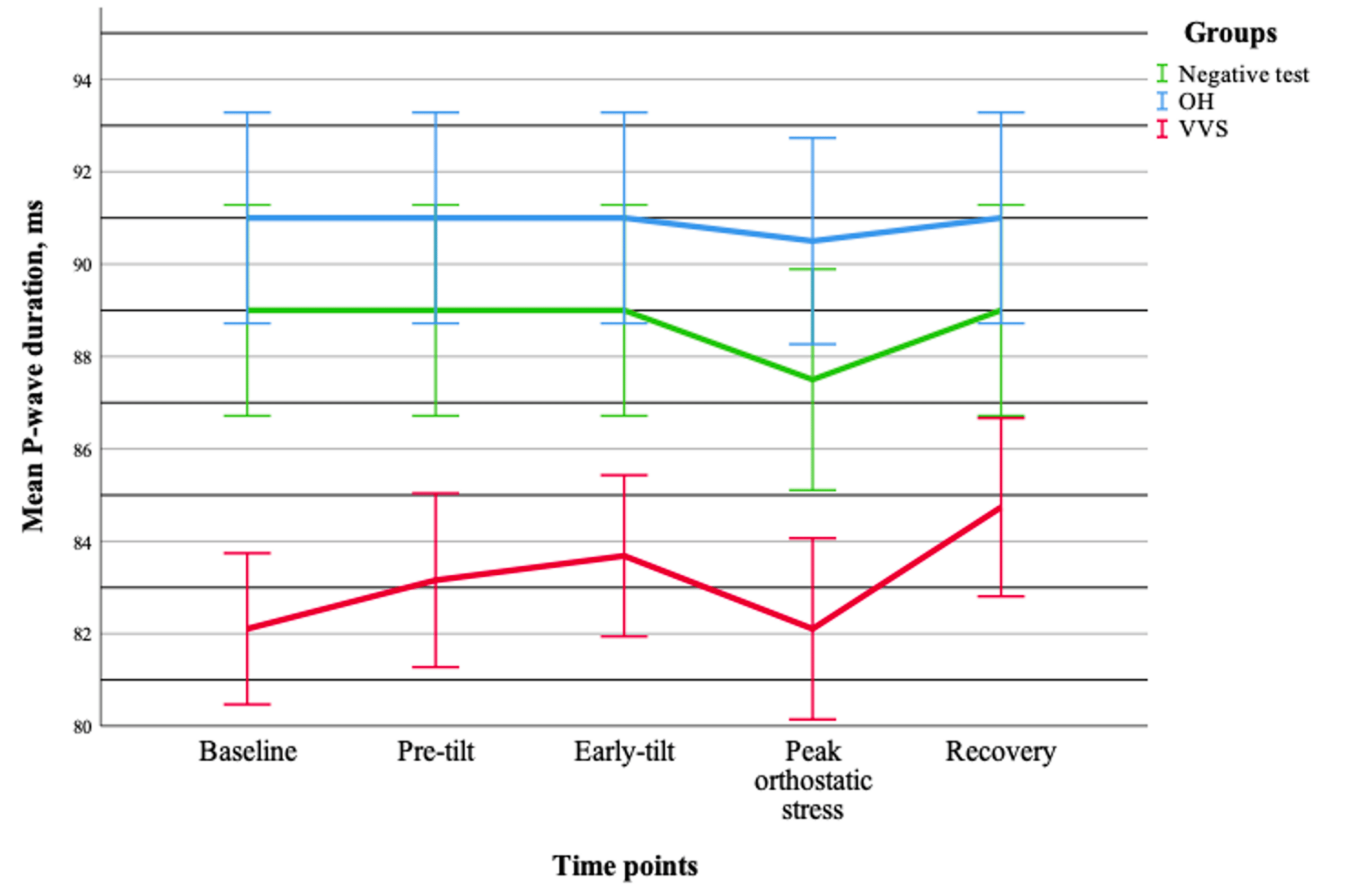
Changes in mean P-wave duration (±standard error) during tilt-table testing across different groups. Data are presented for OH (n = 20), VVS (n = 19), and negative test (n = 20) groups. OH = orthostatic hypotension; VVS = vasovagal syncope

P-wave duration showed no significant interaction between group and time (p = 0.433), indicating a similar pattern of changes across all groups during orthostatic testing. No significant time effect was observed (p = 0.125), suggesting relative stability over the observation period. However, a significant group effect was detected (p = 0.035), demonstrating differences between groups irrespective of time. Post hoc analysis revealed that P-wave duration was significantly longer in the OH group; however, this finding should be interpreted with caution, given the significant age imbalance between groups (Figure 1).

**Figure 2:**
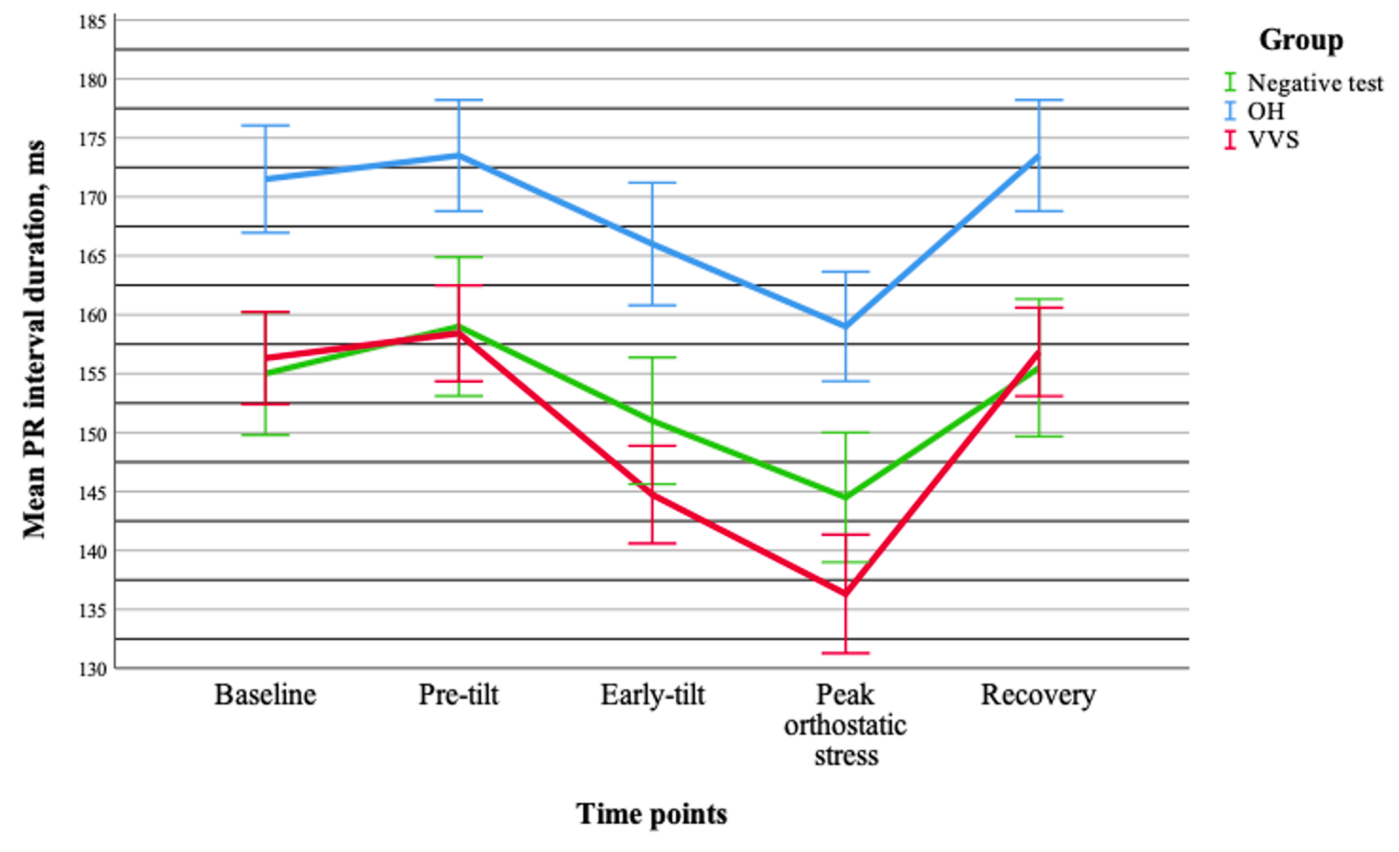
Changes in the mean PR interval duration (±standard error) during tilt-table testing across different groups. Data are presented for OH (n = 20), VVS (n = 19), and negative test (n = 20) groups. OH = orthostatic hypotension; VVS = vasovagal syncope

Similarly, no significant interaction between group and time was found for the PR interval (p = 0.295), indicating comparable temporal patterns across groups. A significant group effect was present (p = 0.015), with longer PR intervals observed in the OH group compared to the VVS group (p = 0.024). In addition, a significant time effect was identified (p < 0.001), reflecting dynamic changes during orthostatic testing. The largest differences were observed between the peak orthostatic response and other time points (p < 0.001) (Figure 2).

**Figure 3:**
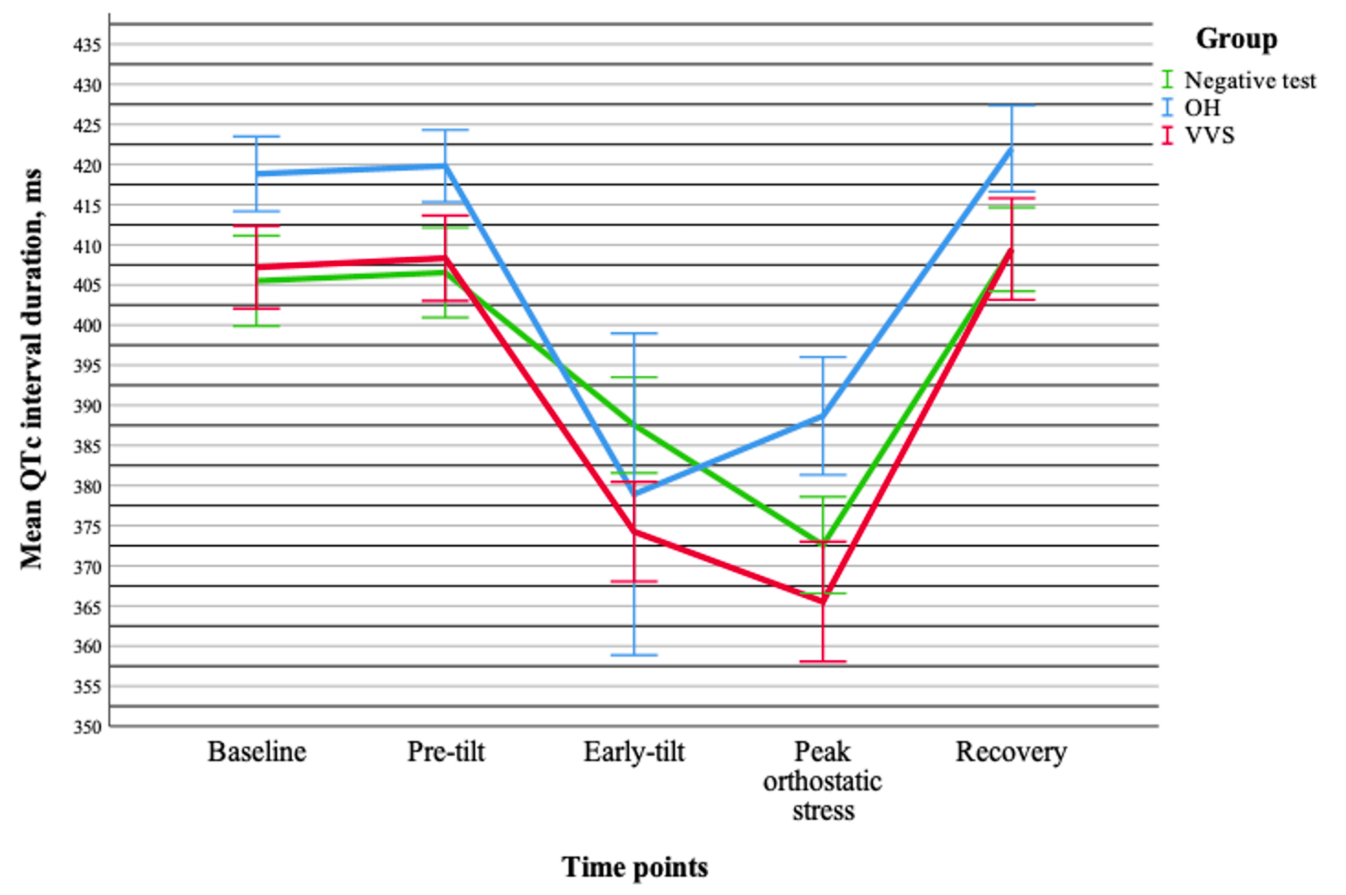
Changes in the mean QTc interval duration (± standard error) during tilt-table testing across different groups. Data are presented for OH (n = 20), VVS (n = 19), and negative test (n = 20) groups. OH = orthostatic hypotension; VVS = vasovagal syncope

QRS duration remained stable, with no significant differences between groups, over time, or in the interaction between these factors (all p > 0.05). In contrast, the QTc interval exhibited significant time-dependent changes (p < 0.001), although no group effect (p = 0.278) or interaction between group and time (p = 0.823) was observed. Pairwise comparisons indicated that the most pronounced differences occurred between the early-tilt and peak orthostatic stress points and in comparisons with other measurements (p < 0.001). No significant differences were found between the baseline and pre-tilt or between the baseline and recovery time points. These findings suggest that QTc changes are most evident during the middle phases of orthostatic testing and are independent of group classification (Figure 3).

**Figure 4:**
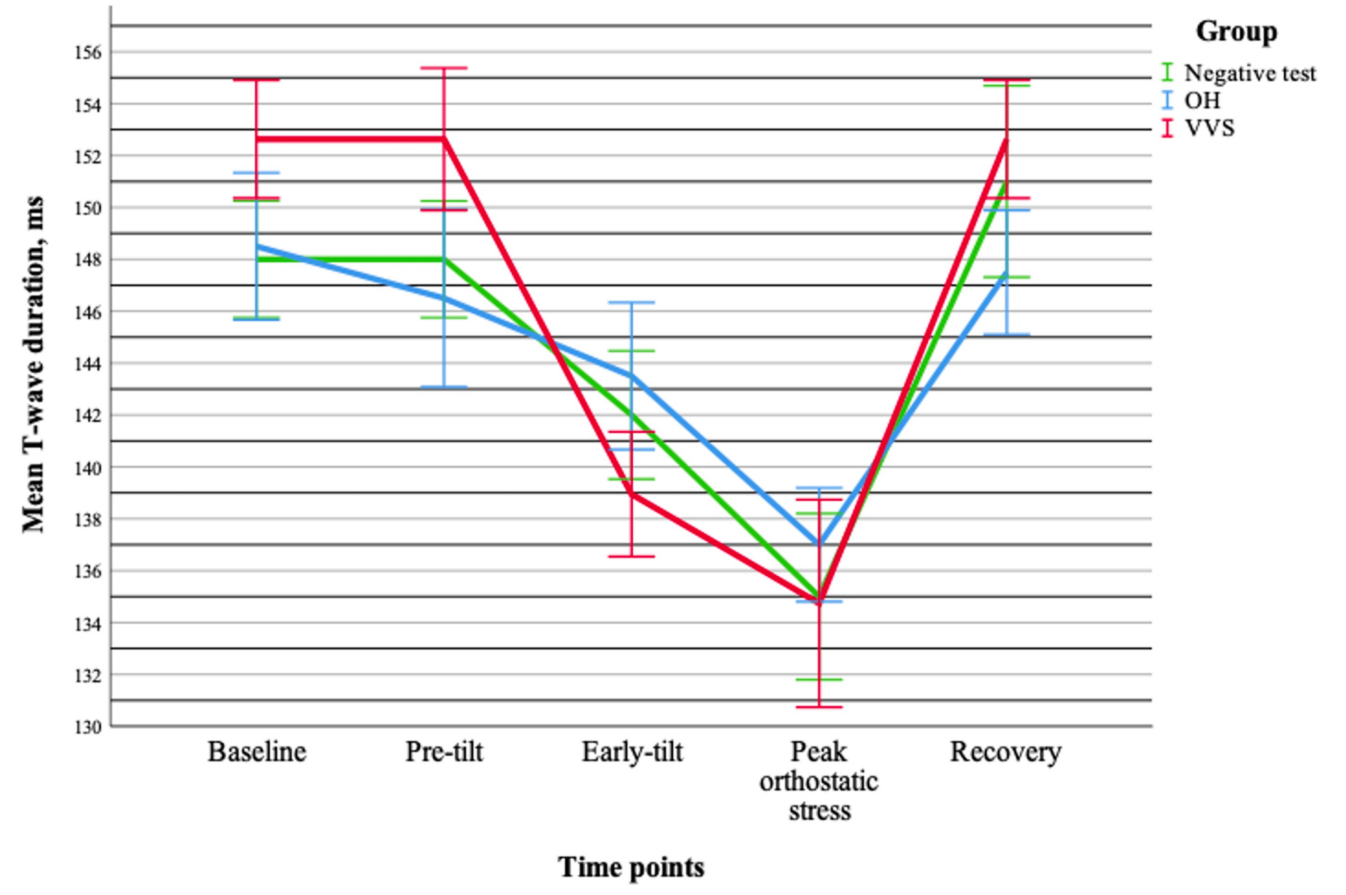
Changes in the mean T-wave duration (±standard error) during tilt-table testing across different groups. Data are presented for OH (n = 20), VVS (n = 19), and negative test (n = 20) groups. OH = orthostatic hypotension; VVS = vasovagal syncope

T-wave duration followed a similar pattern, with no significant group effect (p > 0.05) and no interaction between group and time (p = 0.104), indicating comparable dynamics across groups. However, a significant time effect was observed (p < 0.001), confirming temporal variability. Pairwise comparisons showed that the greatest differences occurred between the middle phases (early-tilt and peak orthostatic stress points) and other measurements (p < 0.001), while no significant differences were found between early and late phases. Mean values demonstrated a decrease in T-wave duration up to the peak orthostatic stress point, followed by a gradual return toward baseline (Figure 4).

**Figure 5:**
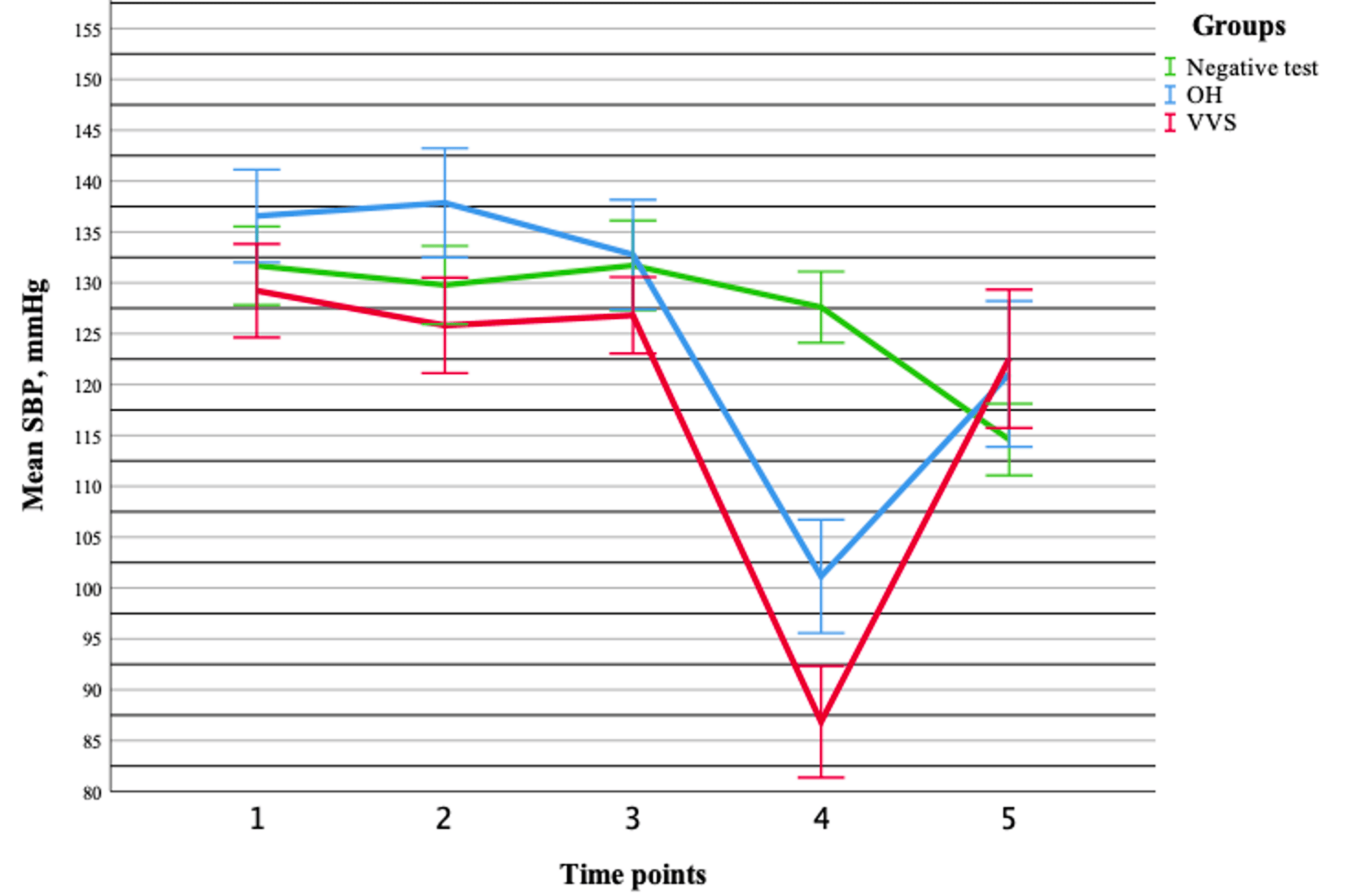
Changes in the mean systolic blood pressure (±standard error) during tilt-table testing across different groups. Data are presented for OH (n = 20), VVS (n = 19), and negative test (n = 20) groups. OH = orthostatic hypotension; VVS = vasovagal syncope; SBP = systolic blood pressure

**Figure 6:**
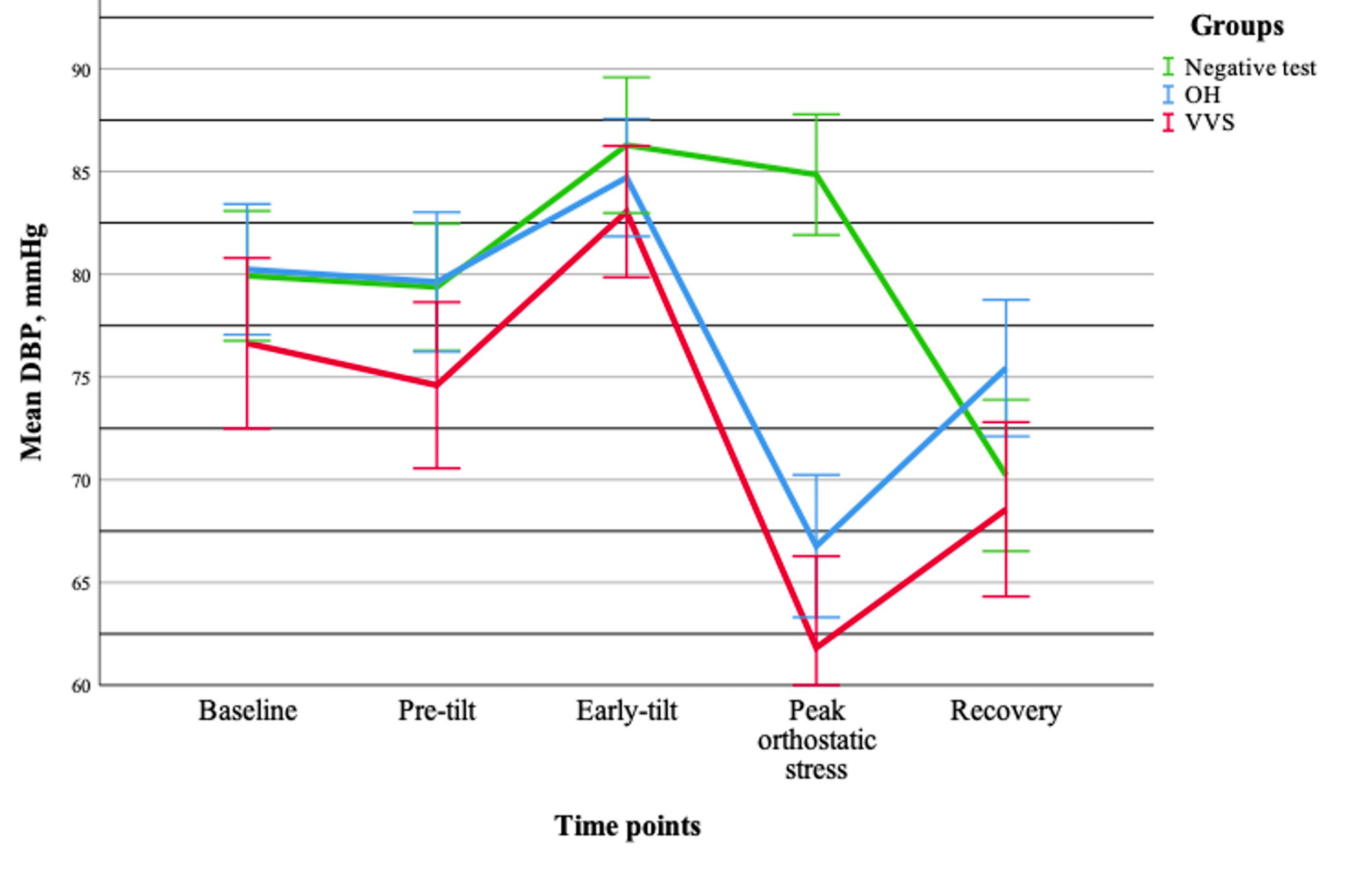
Changes in the mean diastolic blood pressure (±standard error) during tilt-table testing across different groups. Data are presented for OH (n = 20), VVS (n = 19), and negative test (n = 20) groups. OH = orthostatic hypotension; VVS = vasovagal syncope; DBP = diastolic blood pressure

**Figure 7:**
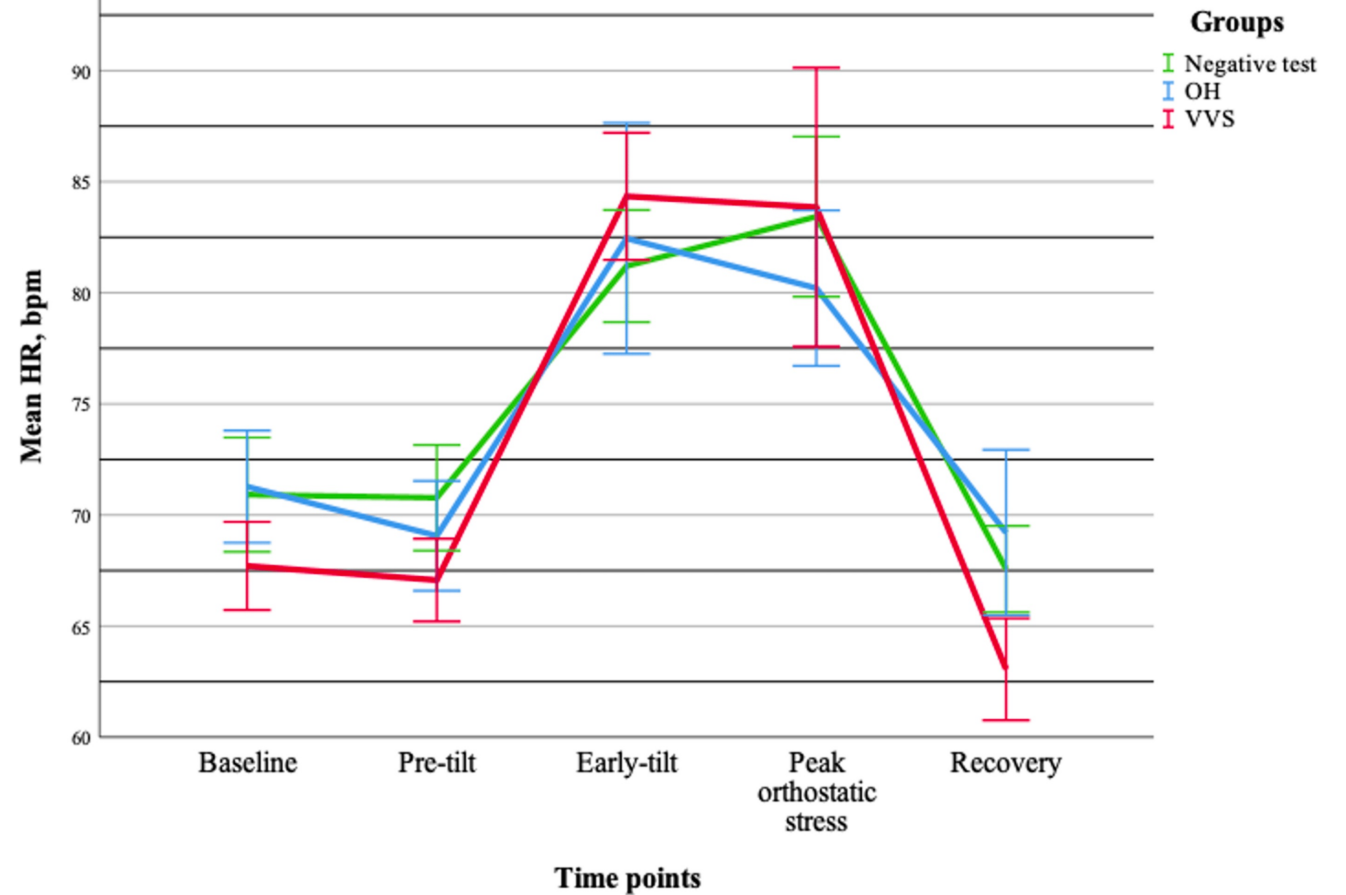
Changes in the mean heart rate (±standard error) during tilt-table testing across different groups. Data are presented for OH (n = 20), VVS (n = 19), and negative test (n = 20) groups. OH = orthostatic hypotension; VVS = vasovagal syncope; HR = heart rate

Assessment of hemodynamic parameters demonstrated that both systolic and diastolic blood pressure changed significantly during orthostatic testing (p < 0.001), without baseline differences between groups. Notably, significant group-time interactions were observed for both systolic (p < 0.001) and diastolic blood pressure (p < 0.001), indicating distinct hemodynamic response patterns across groups. In contrast, heart rate increased significantly over time (p < 0.001) but showed no group effect or interaction (p > 0.05), suggesting a uniform response across all groups. Pairwise comparisons revealed a dynamic blood pressure pattern characterized by an initial compensatory phase, followed by a marked decline during the peak orthostatic phase and subsequent partial recovery. Meanwhile, heart rate demonstrated a sustained increase throughout orthostatic stress. Overall, these findings indicate that blood pressure responses provide greater discriminatory value between syncope types, whereas heart rate reflects a common compensatory autonomic response (Figures 5-7).

A summary of the main ECG and hemodynamic findings is presented in Table 2. Notably, ECG parameters showed limited discriminatory value between groups, whereas hemodynamic parameters, particularly systolic and diastolic blood pressure, exhibited significant group-time interactions, highlighting their greater potential in differentiating syncope mechanisms.

**Table 2:** Summary of electrocardiographic and hemodynamic parameter changes during tilt-table testing. Continuous variables were analyzed using linear mixed models (fixed effects: group, time, and group-time interaction). + indicates statistically significant (p < 0.05), - indicates not statistically significant. SBP = systolic blood pressure; DBP = diastolic blood pressure; HR = heart rate

Parameter	Group effect	Time effect	Interaction (group-time)
P-wave duration	+	-	-
PR interval duration	+	+	-
QRS duration	-	-	-
QTc duration	-	+	-
T-wave duration	-	+	-
SBP	-	+	+
DBP	-	+	+
HR	-	+	-

## Discussion

The present study aimed to evaluate ECG and hemodynamic responses during tilt-table testing and their relevance in differentiating syncope mechanisms. The findings suggest that, although ECG parameters reflect dynamic changes during orthostatic stress, their ability to distinguish between syncope types is limited. In contrast, hemodynamic responses, particularly blood pressure changes, appear to play a more prominent role in differentiating underlying mechanisms.

Among ECG variables, P-wave duration and PR interval differed between groups; however, their temporal patterns during tilt-table testing were similar, with no significant group-time interactions observed. In our study, a significant age difference was observed between the OH and VVS groups (median age = 65 vs. 36 years), which may partly explain the observed differences in PR interval and P-wave duration. It is well established that the PR interval increases with age due to progressive slowing of atrioventricular conduction, as well as structural and functional changes in the cardiac conduction system [11]. Therefore, the longer PR intervals observed in the OH group may be partly explained by age-related physiological changes rather than disease-specific mechanisms. Previous studies have reported that PR interval shortening may occur during tilt-table testing and may be associated with autonomic changes preceding syncope [5,12,13]. Consistent with this, patients with cardioinhibitory syncope have been shown to have similar baseline hemodynamic parameters compared to controls, both at rest and during head-up tilt. However, immediately before syncope, shortening of PR and RR intervals may occur, reflecting dynamic changes in autonomic nervous system activity [14]. Overall, these findings suggest that although baseline differences in atrial conduction and atrioventricular delay may exist, the electrophysiological response to orthostatic stress remains largely similar across different types of syncope.

Furthermore, other ECG parameters, including QTc interval and T-wave duration, showed significant changes over time but did not differ between groups, suggesting that these alterations reflect a generalized physiological response rather than disease-specific mechanisms. Previous studies have reported that T-wave changes and moderate QTc prolongation are relatively common during tilt-table testing, particularly in younger individuals, and are generally considered benign [15]. In contrast, in our study, the QTc interval tended to shorten during orthostatic stress. This discrepancy may be explained by differences in study populations, such as age distribution and autonomic balance, as well as variations in measurement timing and methodology. QRS duration remained stable throughout the test and did not differ between groups, consistent with previous findings that intraventricular conduction is largely unaffected by orthostatic stress [9]. Together, these observations suggest that while ECG parameters reflect global autonomic influences on cardiac electrophysiology, they may have limited specificity in differentiating between syncope mechanisms.

In contrast, hemodynamic parameters demonstrated a markedly different pattern. Both systolic and diastolic blood pressure showed significant group-time interactions, indicating that the dynamics of blood pressure changes during orthostatic testing differ between syncope types. Previous studies have shown that hemodynamic parameters do not significantly differ between patients with positive and negative tilt-table test outcomes in the supine position; however, during orthostatic stress, significant differences, particularly in heart rate and stroke volume, become apparent [16]. Furthermore, VVS has been associated with a sudden hemodynamic collapse, often driven by a rapid reduction in cardiac output and peripheral vascular resistance [17]. This is consistent with our findings, as a more pronounced and abrupt decrease in blood pressure was observed during peak orthostatic stress in the VVS group compared to the OH group. The observed blood pressure patterns, characterized by an initial compensatory phase, followed by a decline during peak orthostatic stress and subsequent partial recovery, align with known mechanisms of orthostatic intolerance and vasovagal responses [18]. Heart rate increased significantly during orthostatic testing but did not differ between groups and showed no significant interaction with time. This suggests that heart rate represents a common compensatory autonomic response to orthostatic stress rather than a distinguishing feature between syncope types. Previous studies have demonstrated that hemodynamic responses during tilt-table testing are closely linked to baroreflex-mediated autonomic control, while additional parameters, such as heart rate variability and end-tidal CO₂, may better reflect symptom severity than heart rate alone [19]. Moreover, different heart rate response patterns have been described depending on the underlying mechanism of syncope. While an appropriate increase in heart rate is expected during upright posture, an impaired or absent response in the presence of blood pressure decline may indicate autonomic dysfunction [20]. Overall, the dissociation between blood pressure and heart rate responses suggests that vascular regulation may play a more critical role than chronotropic responses in differentiating syncope mechanisms.

Overall, the combined analysis of ECG and hemodynamic parameters supports the conclusion that blood pressure responses provide greater clinical value in distinguishing syncope types compared to ECG-derived measurements. While ECG changes reflect generalized autonomic adaptation, they are insufficiently specific for differential diagnosis when used in isolation. Therefore, the integration of hemodynamic data, particularly blood pressure dynamics, with clinical assessment remains essential in the evaluation of patients undergoing tilt-table testing. These findings emphasize that diagnostic assessment should primarily rely on hemodynamic responses rather than ECG parameters alone.

Study limitations

Despite these findings, several limitations of this study should be acknowledged. First, the retrospective design and the selection of patients based on the availability of high-quality ECG recordings may introduce selection bias. Second, the relatively small sample size limits the generalizability of the findings. Third, this was a single-center study without an external validation cohort, which may further restrict the external validity of the results. ECG measurements were performed manually by a single investigator, and interobserver or intraobserver variability was not assessed, which may introduce measurement bias. In addition, QTc was calculated using the Bazett formula, which is known to overcorrect at higher heart rates and may affect the accuracy of QTc estimation. Furthermore, assumptions of the linear mixed models were not formally tested, which may influence the robustness of the findings. An important limitation is the significant age imbalance between groups, which represents a potential confounding factor, particularly for ECG parameters such as the PR interval. As age is known to influence cardiac conduction, the observed differences between groups may be partly explained by age-related physiological changes rather than differences in syncope mechanisms. Moreover, age was not included as a covariate in the statistical models, which may further affect the interpretation of group differences.

## Conclusions

ECG parameters demonstrated significant changes during tilt-table testing; however, they were not sufficiently specific to differentiate between syncope types. In contrast, hemodynamic parameters, particularly systolic and diastolic blood pressure, showed distinct group-specific response patterns and provided greater diagnostic value. Heart rate reflected a common compensatory response and did not contribute to differentiation between groups. These findings suggest that the evaluation of syncope during tilt-table testing may primarily rely on hemodynamic responses rather than ECG parameters.
